# Supplementary figures and images for: Towards the elimination of FGM by 2030: A statistical assessment
Source: PLoS One. 2020 Oct 6;15(10):e0238782. doi: 10.1371/journal.pone.0238782 (PMC7537854; doi:10.1371/journal.pone.0238782)

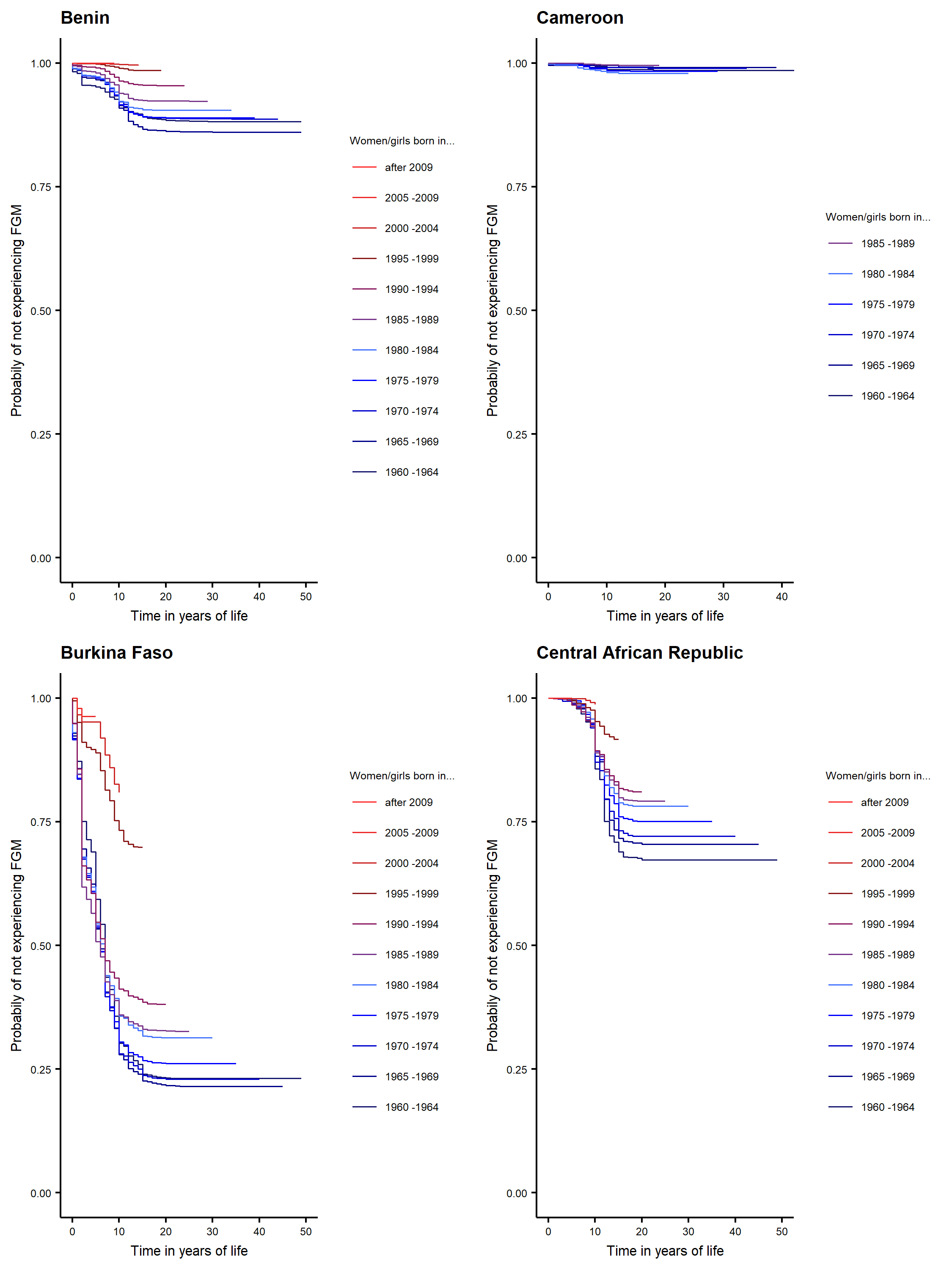

Supplement: S1 Fig — (TIF) [file pone.0238782.s001.tif]

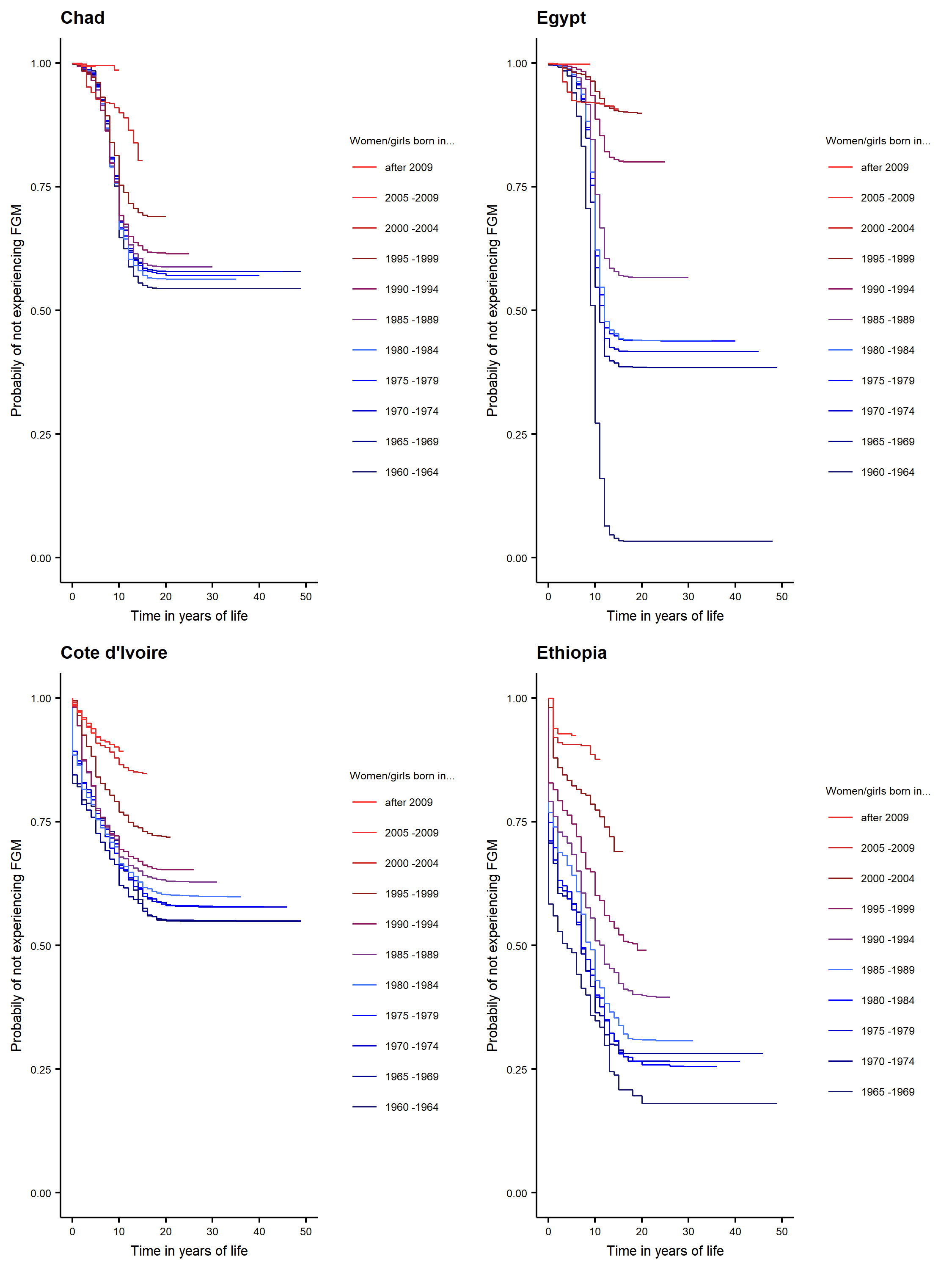

Supplement: S2 Fig — (TIF) [file pone.0238782.s002.tif]

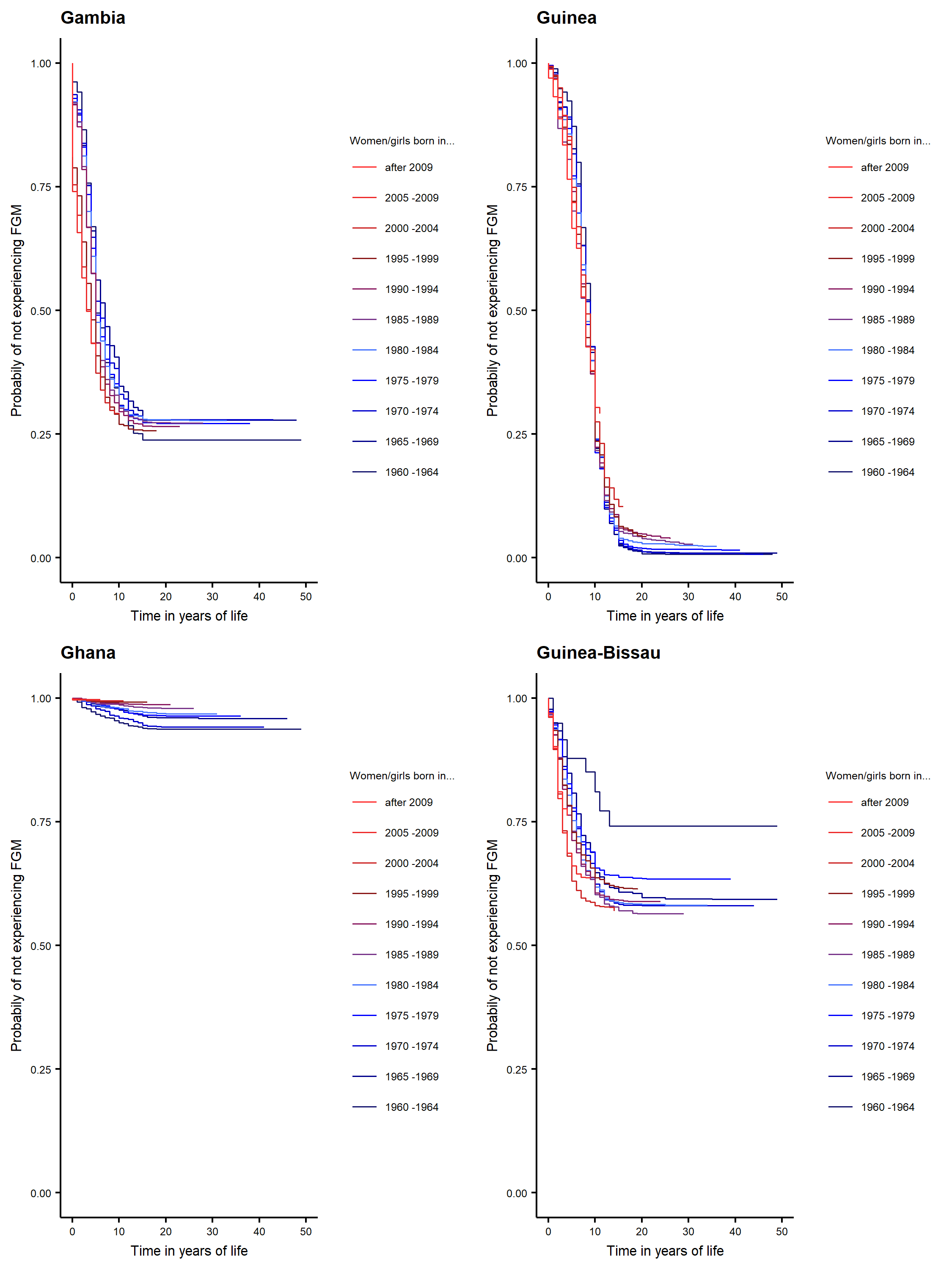

Supplement: S3 Fig — (TIF) [file pone.0238782.s003.tif]

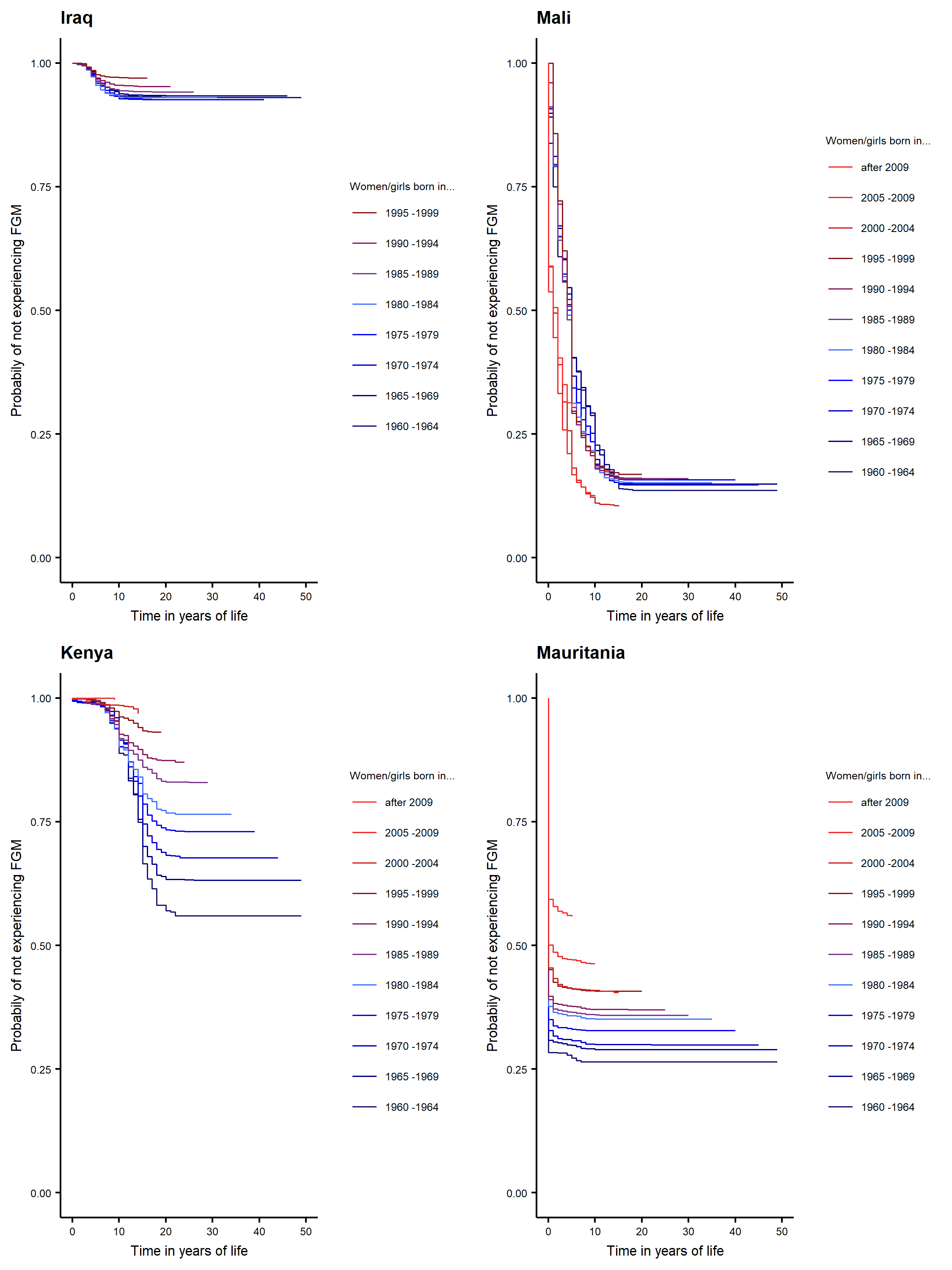

Supplement: S4 Fig — (TIF) [file pone.0238782.s004.tif]

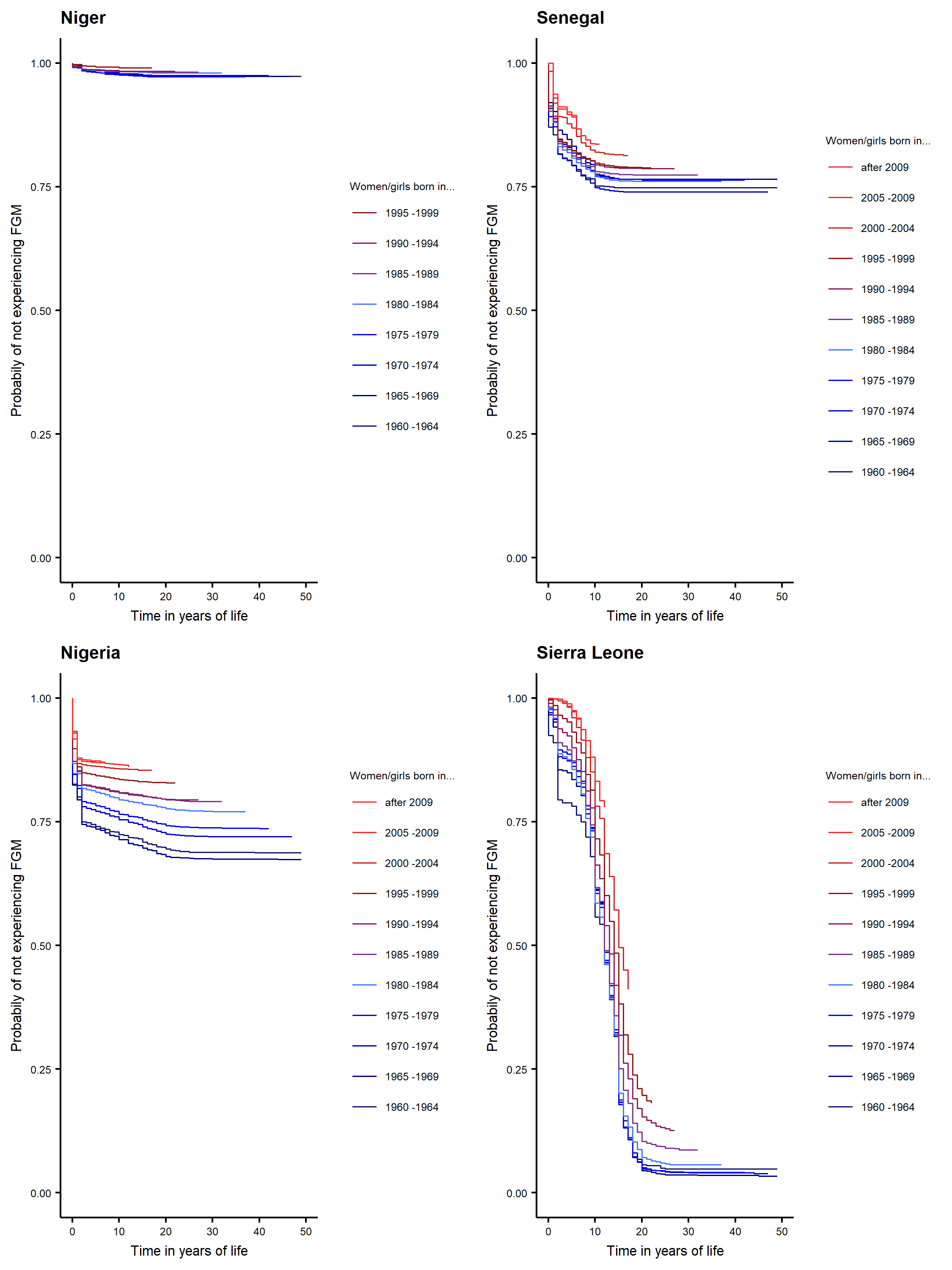

Supplement: S5 Fig — (TIF) [file pone.0238782.s005.tif]

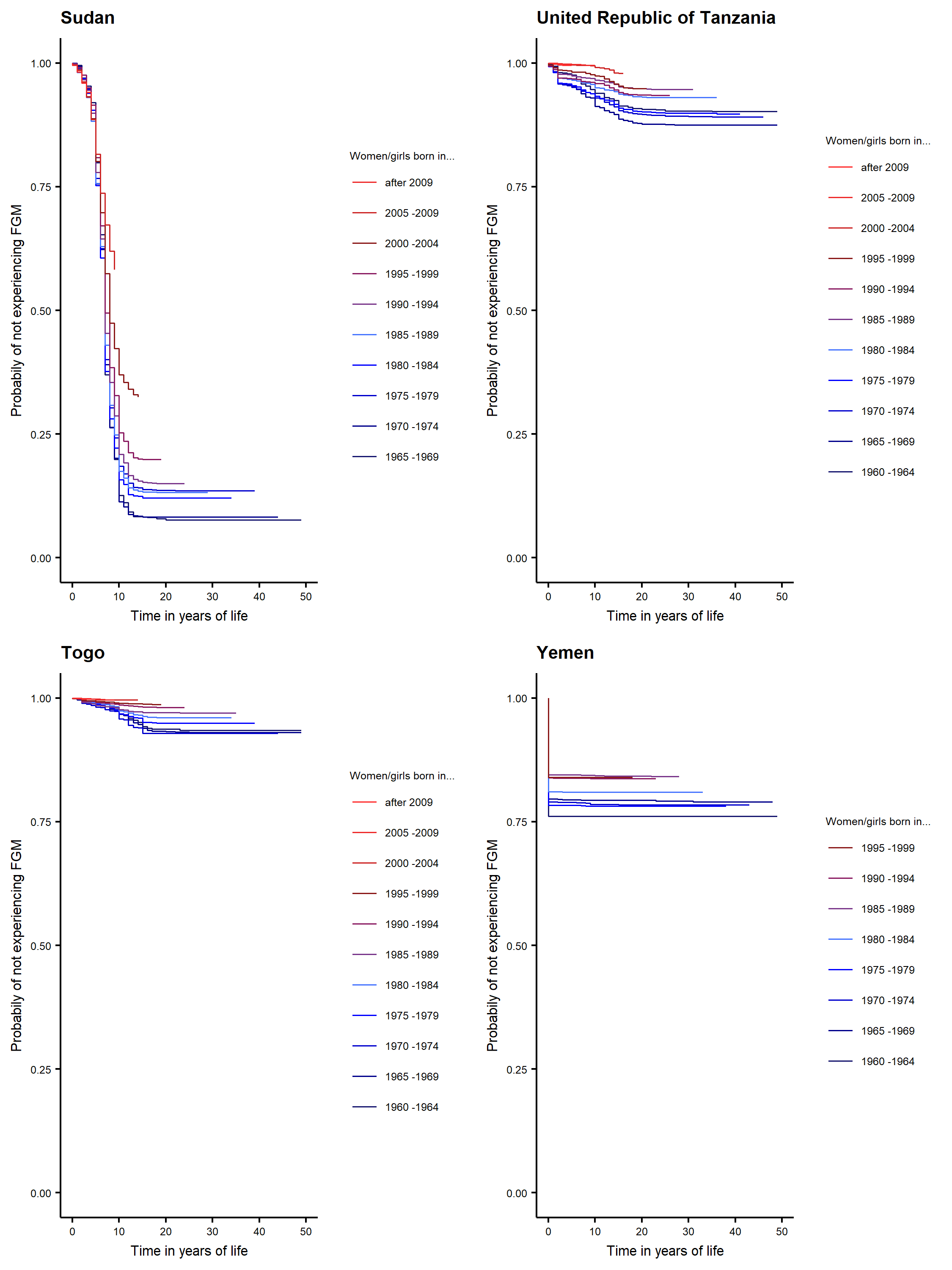

Supplement: S6 Fig — (TIF) [file pone.0238782.s006.tif]
