# Supplementary material for: Towards the elimination of FGM by 2030: A statistical assessment
Source: PLoS One. 2020 Oct 6;15(10):e0238782. doi: 10.1371/journal.pone.0238782 (PMC7537854; doi:10.1371/journal.pone.0238782)
Supplement: S1 Appendix — (DOCX) [file pone.0238782.s007.docx]

**S1 Appendix. Data exclusions**

Excluded regions and surveys due to inconsistencies in coverage include:

- The region ‘Vakaga’ which was covered in the 1994/1995 DHS in Central African Republic had to be dropped as it was excluded in the 2010 MICS [1].
- Egypt’s DHS’ in 1994, 2005, and 2014 mothers’ module had to be excluded from the analysis due to changes in sampling. Until 2008 and in 2014, only ever-partnered women were surveyed [2, 3].
- In addition, North and South Sinai had to be dropped from the 2008 DHS in Egypt, as these regions could not be accessed in later surveys [2].
- The ‘North-Eastern’ region in Kenya was excluded 2008/09 DHS and 2014 DHS, as it had not been surveyed in the 1998 DHS [4].
- Mali 2013 DHS was not nationally representative due to the exclusion of Gao, Tombouctou, Kidal and three areas in Mopti [5].
- The two surveys conducted in 2011 on Somalian territory have been excluded as they were not nationally representative.
- Senegal 2013 DHS had to be excluded as only daughters’ module was included.

**References**

1. Comité National de Lutte contre le VIH SIDA, WHO, FAO, UNFPA, UNICEF, World Bank, ICF International. Enquête par grappes à indicateurs multiples – MICS couplée avec la sérologie VIH, RCA. 2010. Bangui, Central African Republic: L’Institut Centrafricain des Statistiques, et des Etudes Economiques et Sociales, UNICEF, UNFPA. Available from : <http://mics.unicef.org/surveys> French.
2. El-Zanaty F, Way A. Egypt Demographic and Health Survey 2008. Cairo, Egypt: Ministry of Health [Egypt], El-Zanaty and Associates, and Macro International:2009. Available from: <https://dhsprogram.com/pubs/pdf/FR220/FR220.pdf>
3. Ministry of Health and Population [Egypt], El-Zanaty and Associates, and ICF International. Egypt Demographic and Health Survey 2014. Cairo, Egypt: Ministry of Health and Population and ICF International: 2015. Available from: <https://dhsprogram.com/pubs/pdf/fr302/fr302.pdf>
4. National Council for Population and Development (NCPD), Central Bureau of Statistics (CBS) (Office of the Vice President and Ministry of Planning and National Development), and Macro International Inc. Kenya Demographic and Health Survey 1998. 1999 Apr. Calverton, Maryland: Available from: <https://dhsprogram.com/pubs/pdf/FR102/FR102.pdf>
5. Cellule de Planification et de Statistique (CPS/SSDSPF) [Mali], Institut National de la Statistique (INSTAT/MPATP) [Mali], INFO-STAT et ICF International. Enquête Démographique et de Santé au Mali 2012-2013. Rockville, Maryland, USA:CPS, INSTAT, INFO-STAT et ICF International: 2014. Available from: <https://dhsprogram.com/pubs/pdf/FR286/FR286.pdf> French.
