# Supplementary material for: Towards the elimination of FGM by 2030: A statistical assessment
Source: PLoS One. 2020 Oct 6;15(10):e0238782. doi: 10.1371/journal.pone.0238782 (PMC7537854; doi:10.1371/journal.pone.0238782)
Supplement: S1 Table — (DOCX) [file pone.0238782.s008.docx]

S1 Table outlines the data sources by survey, including the percentage of women who reported that they could not remember the exact age at which they experienced FGM. If a woman could remember to experience, FGM early on but only vaguely recalled when her daughter was cut, or when she was cut, DHS and MICS typically code this as cut ‘During infancy’. In this case, the survey reports most commonly define which age range is implied. We have not systematically used these responses, as due to the larger recall bias in older cohorts, a strict exclusion would bias our results. The exact age-at-FGM was estimated by applying the same distribution as observed among women who could remember the exact year of FGM in the same survey. In the case of Mauritania, “during infancy” was estimated as at age 0, as implied by daughter cohorts of the same survey, and responses from other surveys for Mauritania.

|  | **Survey** | **Type** | **% of women (15-49 years) answering “During infancy” (unweighted)** |
| --- | --- | --- | --- |
| **1** | **Benin 2001** | DHS | 30.4% |
| **2** | **Benin 2006** | DHS | 46.2% |
| **3** | **Benin 2011-12** | DHS | 26.1% |
| **4** | **Benin 2014** | MICS | - |
| **5** | **Burkina Faso 1998/99** | DHS | - |
| **6** | **Burkina Faso 2003** | DHS | 40.7% |
| **7** | **Burkina Faso 2010** | DHS | 46.0% |
| **8** | **Cameroon 2004** | DHS | 12.1% |
| **9** | **CAR 1994/95** | DHS | 0.2% |
| **10** | **CAR 2010** | MICS | - |
| **11** | **Chad 2000** | MICS | - |
| **12** | **Chad 2004 *** | DHS | 43.8% |
| **13** | **Chad 2010** | MICS | - |
| **14** | **Chad 2014-15** | DHS | 6.4% |
| **15** | **Côte d'Ivoire 1994** | DHS | - |
| **16** | **Côte d'Ivoire 1998-99 **** | DHS | 51.8% |
| **17** | **Côte d'Ivoire 2011-12** | DHS | 47.5% |
| **18** | **Côte d'Ivoire 2016** | MICS | - |
| **19** | **Egypt 2008** | DHS | - |
| **20** | **Egypt 2014 (only daughters’ module)** | DHS | - |
| **21** | **Egypt 2015** | DHS | - |
| **22** | **Ethiopia 2016** | DHS | 41.3% |
| **23** | **Gambia 2010** | MICS | - |
| **24** | **Gambia 2013** | DHS | 0.3 |
| **25** | **Ghana 2011** | MICS | - |
| **26** | **Guinea 1999** | DHS | - |
| **27** | **Guinea 2005** | DHS | 36.1% |
| **28** | **Guinea 2012** | DHS | 21.4% |
| **29** | **Guinea 2016** | MICS | - |
| **30** | **Guinea-Bissau 2014** | MICS | - |
| **31** | **Iraq 2011** | MICS | 0.3% |
| **32** | **Kenya 1998** | DHS | - |
| **33** | **Kenya 2008-09** | DHS | 1.9% |
| **34** | **Kenya 2014** | DHS | 1.5% |
| **35** | **Mali 1995/96** | DHS | - |
| **36** | **Mali 2001** | DHS | 48.6% |
| **37** | **Mali 2006** | DHS | 59.7% |
| **38** | **Mali 2009/10** | MICS | 66.4 |
| **39** | **Mali 2015** | MICS | 60.3% |
| **40** | **Mauritania 2011** | MICS | 91.6% |
| **41** | **Mauritania 2015** | MICS | - |
| **42** | **Niger 1998** | DHS | - |
| **43** | **Niger 2006** | DHS | 54.5% |
| **44** | **Niger 2012** | DHS | 59.1% |
| **45** | **Nigeria 2003** | DHS | 68.3% |
| **46** | **Nigeria 2008** | DHS | 77.6% |
| **47** | **Nigeria 2011** | MICS | 56.8% |
| **48** | **Nigeria 2013** | DHS | 76.6% |
| **49** | **Nigeria 2017** | MICS | - |
| **50** | **Senegal 2005** | DHS | 63.9% |
| **51** | **Senegal 2010-11** | DHS | 52.2% |
| **52** | **Senegal 2014** | DHS | 55.8% |
| **53** | **Senegal 2015** | DHS | 55.5% |
| **54** | **Senegal 2016** | DHS | 60.4% |
| **55** | **Senegal 2017** | DHS | 69.7% |
| **56** | **Sierra Leone 2008** | DHS | 20.7% |
| **57** | **Sierra Leone 2010** | MICS | 0.0% |
| **58** | **Sierra Leone 2013** | DHS | 14.7% |
| **59** | **Sierra Leone 2017** | MICS | - |
| **60** | **Sudan 2014** | MICS | - |
| **61** | **Tanzania 1996** | DHS | - |
| **62** | **Tanzania 2004-05** | DHS | 29.6% |
| **63** | **Tanzania 2010** | DHS | 36.0% |
| **64** | **Tanzania 2015-16** | DHS | 37.2% |
| **65** | **Togo 2010** | MICS | 15.8% |
| **66** | **Togo 2013-14** | DHS | 23.5% |
| **67** | **Yemen 2013** | DHS | 0.5 |
|  | **** before 5, between 5-9, after 10*** | |  |
|  | ***** early neonatal*** | |  |

# 
