# Supplementary material for: Towards the elimination of FGM by 2030: A statistical assessment
Source: PLoS One. 2020 Oct 6;15(10):e0238782. doi: 10.1371/journal.pone.0238782 (PMC7537854; doi:10.1371/journal.pone.0238782)
Supplement: Attachment — Submitted filename: Response to Reviewers.docx [file pone.0238782.s009.docx]

Dear Sir or Madam,

We would like to thank PlosOne’s academic editor and the peer reviewers for their helpful comments and suggestions with respect to our manuscript as well as providing us with the opportunity to submit a revision.

The style requirements, including file names, positioning of abstracts as well as title of the manuscript have been amended according to the style requirements of PlosOne. The updated version of our manuscript has been uploaded in the submission form, as well as in the ‘Revised Manuscript with Track Changes’.

Below we have drafted responses to all comments and questions by the reviewers listing amendments we have made within the manuscript.

**Reviewer 1:**

I. **On the definition of risk of FGM**

We have included a particular note in the introduction outlining the definition of ‘risk of FGM’ as one of experiencing FGM at a given age IF one has not experienced FGM earlier in life. This provides a more explicit definition compared with the previous version. This note can be found in the ‘Introduction and background’ section.

II. **On the inclusion criteria of DHS/MICS surveys**

With respect to this question, we would like to note that ‘S7- Appendix. Data exclusions describes surveys that are not part of the analysis including the exclusion criteria. As per recommendations by the reviewers, we have also added the main exclusion criteria to the main body of the text in the ‘Materials and methods’ section.

III. **On the distinction between DHS/MICS before 2010 and after**

S8 Table in the Annex describes each single survey including survey year and number of responses that are ‘during infancy’. The cut-off between surveys that included a module on daughters cannot be strictly made in the year 2010, as countries, such as Iraq, do not include a daughter module, even after 2010. Therefore, the full list of countries in S8 - Table is necessary for complete transparency and has now been added in the appendix.

IV. **On the inclusion of analysis of the type of FGM**

The type of FGM is indeed included in the vast majority of FGM survey modules both for DHS and MICS. However, we have not included an analysis of type of FGM in this analysis and estimated the overall risk of FGM of all types for several reasons.

First and foremost, entrenched gender inequality is behind all types of FGM, independent of their invasiveness. In addition, all forms of FGM are associated with increased health risk and violates the sexual and reproductive health and rights of women and girls.

With the adoption of the Sustainable Development Goals, the UN and its member states have pledged to eliminate the practice by 2030, in all types and forms, and our analysis is providing a background evaluation of the feasibility of this pledge. Zero tolerance to all forms of female genital mutilation is the guiding principle of this analysis.

V. **On the inclusion of analysis regarding medicalization of FGM**

It is true that medicalization of female genital mutilation (practice of female genital mutilation by health care providers) is an alarming trend which requires due attention. Medicalization of female genital mutilation is more common in certain countries and programmes need to respond to this context in these countries. However, discussion on medicalization is beyond the scope of this article. For the article, the main emphasis is to project the number of girls at risk of female genital mutilation based on population data instead of going into details of who performs the practice.

VI. **On the inclusion of analysis on anti-FGM legislation**

This is a very valuable comment with respect to reliability of survey results retrieved from self-reporting by women, and proxy reporting of women with respect to their daughters.

We have included that validation analysis is of particular importance given the recall bias as well as the changing landscape of national legal frameworks in paragraph 9 of the discussion section.

We feel that given the other potential sources of biases, such as the self-reporting for women and the proxy reporting for girls, the different age groups and the extent of the recall bias, among others, the point is covered with due justice and further elaboration is beyond the scope of the current analysis.

VII. **On the inclusion of more language connecting the current analysis and the projection for elimination of FGM by 2030**

This is a very helpful comment, and we have added a further explanation in paragraphs 4 and 10 of the discussion section on the meaning of the results of our analysis for the probability of the global elimination of FGM by 2030.

VIII. **On the question on improving FGM data collection, processing and presentation**

We have added more details with respect to increased data collection efforts beyond DHS/MICS that would be necessary to estimate FGM trends (Please see paragraphs 6 and 9 of discussion section). However, it is important to emphasize that there has been improvement in the questionnaire used by DHS/MICS (such as having a module to report on the prevalence for 10-14 years of old) and trend analysis is becoming possible for different countries as there are different data sets from DHS/MICS and through cohort approaches.

We have added further possible expansion of quantitative data sources for the estimation of FGM in the ‘Discussion’ section, such as the increased use of administrative records.

**Reviewer 2:**

IX. **On the inclusion of data form the Asia and Pacific region, and in particular Indonesia**

We agree with the inclusion of this academic resource to enhance our discussion section (paragraph 6 of discussion section). In this section, we maintain that in order that countries in which FGM has been reported anecdotally have to be included in ‘truly global level and trend estimates’, and the suggested literature review on FGM in Asia and the Pacific is a meaningful addition (paragraph 9 of discussion section).

Indeed, Indonesia has conducted a national survey in 2013 including information on the FGM for girls age 0-11. The current analysis, however, required micro data in order to be able to perform the estimations.

We amended the relevant reference in the text to make clear that the lack of data in the case of Indonesia refers to the lack of publicly available micro datasets, not published prevalence estimates in general (paragraph 6 of discussion section).

With kind regards,

Kathrin Weny, Romesh Silva, Rachel Snow, Berhanu Legesse, and Nafissatou Diop
